# Clinicopathologic characteristics and prognosis of synchronous colorectal cancer: a retrospective study

**DOI:** 10.1186/s12876-022-02153-9

**Published:** 2022-03-13

**Authors:** Huaxian Chen, Shi Yin, Zhizhong Xiong, Xianzhe Li, Fengxiang Zhang, Xijie Chen, Jianping Guo, Minghao Xie, Chaobin Mao, Longyang Jin, Lei Lian

**Affiliations:** 1 Department of Gastrointestinal Surgery, The Sixth Affiliated Hospital, Sun Yat-sen University, 26 Yuancun Er Heng Rd, Guangzhou, Guangdong China; 2 Guangdong Institute of Gastroenterology, Guangdong Provincial Key Laboratory of Colorectal and Pelvic Floor Diseases, The Sixth Affiliated Hospital, Sun Yat-sen University, Guangzhou, China; 3 Department of General Surgery, The First Affiliated Hospital, Nanchang University, Nanchang, China

**Keywords:** Colorectal cancer, Synchronous colorectal cancer, Early-onset colorectal cancer, Prognosis

## Abstract

**Background:**

The clinical characteristics of synchronous colorectal cancer (SCRC) reported in previous studies differ significantly. Furthermore, little is known about the characteristics of early-onset synchronous colorectal cancer (EO-SCRC). The aim of this retrospective study was to identify the clinicopathological characteristics of SCRC and EO-SCRC and define their relevant prognostic factors.

**Methods:**

Patients who underwent surgery for SCRC and primary unifocal colorectal cancer (PCRC) between January 2007 and December 2020 were included in this study. The clinical, histological, and molecular characteristics of the patient’s tumours were analysed. The primary endpoint was overall survival (OS). Univariate and multivariate Cox regression analyses were used to assess the association between clinicopathological factors and patient survival.

**Results:**

A total of 1554 patients were included in the analysis. Of these, 1132 (72.84%) had PCRC and 422 (27.16%) had SCRC. SCRC occurred more frequently in the elderly (*P* < 0.001) and in male patients (*P* = 0.002). The 5-year OS rate was 73.7% ± 2.0% for PCRC and 61.9% ± 3.9% for SCRC (*P* < 0.05). However, the Cox regression analysis showed that SCRC was not an independent prognostic factor for the prediction of OS. A total of 64 patients (15.17%) in the SCRC group had early-onset colorectal cancer (EOCRC), whereas 257 (22.70%) in the PCRC group had EOCRC (*P* = 0.001). The proportion of patients with deficient mismatch repair proteins (dMMR) in EO-SCRC subgroup was significantly higher than that in late-onset synchronous colorectal cancer (LO-SCRC) subgroup (23.44% vs. 10.34%, *P* = 0.006). Patients with EO-SCRC had more TNM stage IV (*P* < 0.001) and fewer opportunities for radical surgery (79.69% vs. 92.22%, *P* = 0.007) than those with early-onset primary unifocal colorectal cancer (EO-PCRC). There was no significant difference in 5-year OS between the EO-SCRC and LO-SCRC subgroups (*P* = 0.091) and between the EO-SCRC and EO-PCRC subgroups (*P* = 0.094). Multivariate analysis revealed that EOCRC was an independent good prognostic parameter for colorectal cancer (CRC) and SCRC.

**Conclusion:**

For patients with operative treatment, EO-SCRC is different from LO-SCRC and EO-PCRC. Patients with SCRC show a poorer survival rate than those with PCRC. However, SCRC is not an independent prognostic factor for CRC, whereas EOCRC is a good prognostic factor for CRC and SCRC.

## Background

Colorectal cancer (CRC) is one of the most common malignant tumours and accounts for approximately 10% of all cancers diagnosed annually [1]. Synchronous colorectal cancer (SCRC), which is a specific type of CRC, refers to the presence of more than one primary colorectal carcinoma in a single individual simultaneously or within six months after surgery for the index cancer. The incidence of SCRC ranges from 1.1 to 8.1% [2]. Due to its low incidence compared with other types of CRCs, large-sample research on SCRC is currently limited. A few relatively small retrospective studies have shown that SCRC has several clinical features that differ from those of primary unifocal colorectal cancer (PCRC), especially in terms of tumour location and pathology [3, 4]. However, the results of these studies are controversial, and a consensus has not yet been reached. Furthermore, there are differences in the molecular biology of SCRC and PCRC that can impact therapeutic outcomes in targeted therapy and tumour immunotherapy [5]. Therefore, the unique clinical and molecular characteristics of SCRC may lead to differences in prognoses. Nevertheless, the differences in clinical characteristics and outcomes between the two types of CRC are still controversial.

CRC is primarily detected in elderly patients with a median age of 66 years at diagnosis [6]. According to an epidemiological survey, the incidence of CRC has been increasing over the past decades, with its incidence in young people showing a gradual upward trend [7]. Although the concept of early-onset colorectal cancer (EOCRC) has been proposed and has been used to describe the occurrence of CRC in young adults, there is no uniform age criterion for EOCRC [8–11]. EOCRC, which has a distinct aetiology and biological characterisation, is explicitly addressed as a unique type of CRC. Previous studies have indicated that EOCRC is associated with a higher percentage of SCRC [12, 13]. The clinicopathologic features related to EOCRC generally present as more invasive lesions, which tend to be poorly differentiated and located in the left colon and the rectum [9]. Moreover, microsatellite instability (MSI) is relatively common in EOCRC [12].

Considering the paucity of research on the clinicopathologic features of these abovementioned subtypes of CRC, we conducted a retrospective analysis of patients with SCRC who received surgical treatment at our institution between 2007 and 2020. In addition, we compared the differences between the clinical features of patients with SCRC and those with PCRC to identify the clinical and molecular characteristics of SCRC. Furthermore, particular attention was paid to the features of early-onset synchronous colorectal cancer (EO-SCRC).

## Methods

### Patients

This retrospective study was conducted to identify the clinicopathological characteristics of SCRC and EO-SCRC and define their relevant prognostic factors. In this retrospective study, the requirement for informed consent was waived, and approval was granted by the Ethical Committee of the Sixth Affiliated Hospital of Sun Yat-Sen University. Four hundred twenty-two patients with SCRC and 1132 patients with PCRC who underwent surgery at the Sixth Affiliated Hospital of Sun Yat-Sen University from January 2007 to December 2020 were included in the study cohort.

### Inclusion and exclusion criteria

**Fig. 1:**
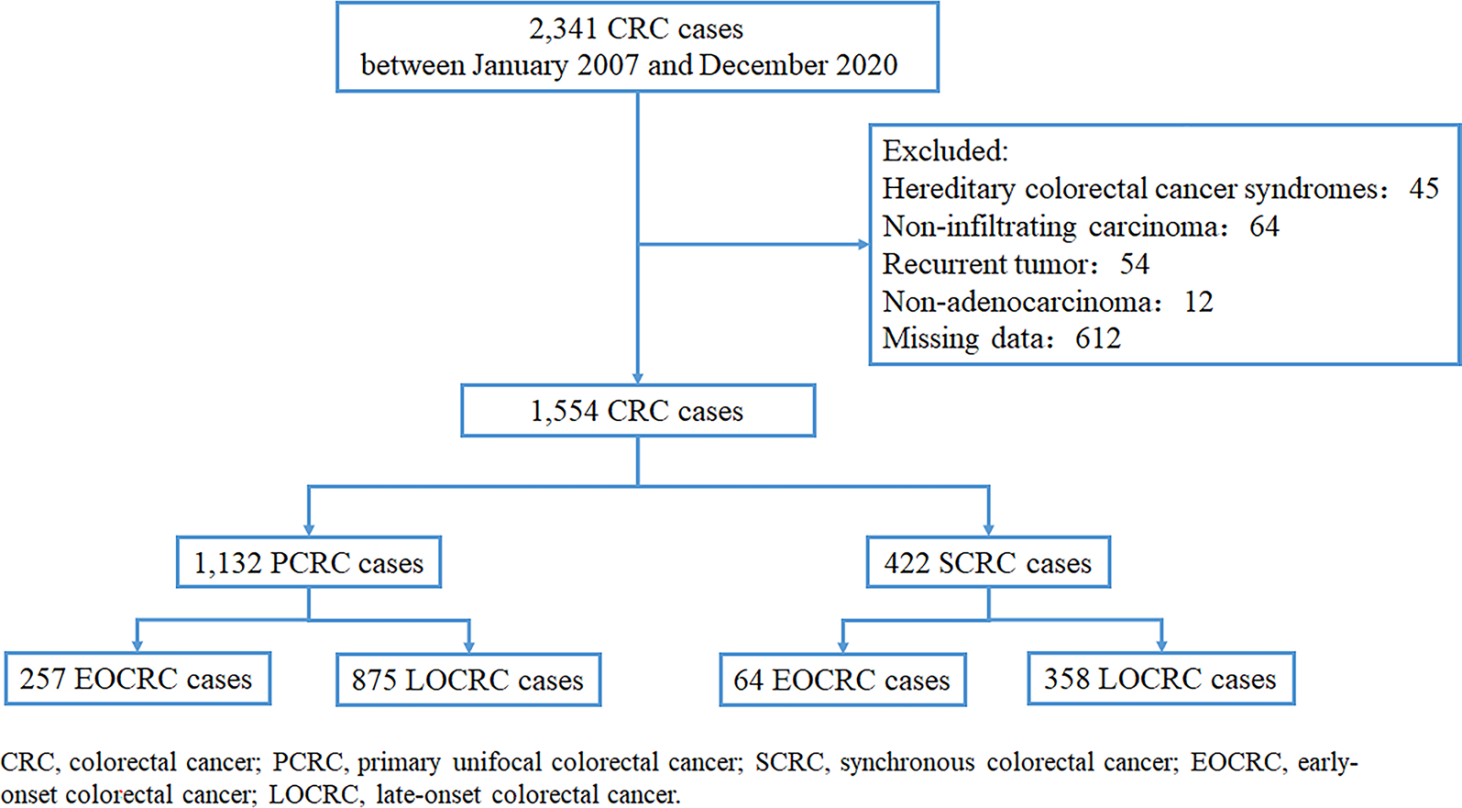
Flow chart of the study

All patients included in the study had complete clinicopathological and follow-up data. All the included patients had infiltrating carcinoma and had undergone surgery. For patients with SCRC, each lesion was not a recurrence or metastasis of another lesion. Patients with any recurrent tumour, familial adenomatous polyposis, or hereditary nonpolyposis colorectal cancer were excluded. A flowchart of the patient selection process is shown in Fig. 1.

### Outcome measures

Clinicopathological data, including age, sex, body mass index (BMI), preoperative serum biochemical indices, tumour location, type of ascites, history of radical or palliative surgery, pathological features, family history of tumour, and mismatch repair protein (MMR) status, were collected from the cancer database of Sixth Affiliated Hospital of Sun Yat-Sen University. Follow-up data were collected from the hospital’s follow-up office.

The patients were classified into two groups for comparison according to the tumour type: PCRC and SCRC groups. The two groups were further divided according to age for subgroup analysis. Multiple primary colorectal carcinomas were defined according to the following criteria: (1) each colorectal tumour must be a histologically-proven malignant lesion and must be an independent and infiltrating lesion; and (2) each lesion is not a recurrence or metastasis of another lesion. SCRC was defined as all colorectal cancers detected simultaneously or within a period of less than six months [14]. The most extensive tumour according to the (y)pTNM staging system was designated as the index tumour. According to the consensus, EOCRC is defined as CRC in patients < 50 years old, whereas late-onset colorectal cancer (LOCRC) is CRC in patients > 50 years old [11]. The primary tumour location was categorised into three groups [15]: (1) right colon, which includes the cecum, ascending colon, and transverse colon; (2) left colon, which includes the splenic flexure, descending colon, and sigmoid colon; and (3) the rectum. Type of ascites was classified as no ascites, serous ascites, and bloody ascites.

BMI was categorised according to the reference standard for Chinese populations (< 18.5 kg/m^2^ vs. 18.5 kg/m^2^ to 24 kg/m^2^ vs. ≥ 24 kg/m^2^) [16]. The cut-off values for haemoglobin (≤ 110 g/L vs. > 110 g/L), platelet (≤ 300 × 10^9^/L vs. > 300 × 10^9^/L), and white blood cell (WBC) (≤ 10 × 10^9^/L vs. > 10 × 10^9^/L) counts were determined as described in the literature [17, 18]. Preoperative serum biochemical indices, including carcinogenic antigen (CEA) (≤ 10 ng/mL vs. > 10 ng/mL), alpha-fetoprotein (AFP) (≤ 7 ng/mL vs. > 7 ng/mL), cancer antigen (CA)19-9 (≤ 27 kU/L vs. > 27 kU/L), CA125 (≤ 35 kU/L vs. > 35 kU/L), and CA153 (≤ 25 kU/L vs. > 25 kU/L) levels, were categorised according to the findings of previous studies [18, 19].

The pathological features assessed included gross tumour classification, histological type, tumour differentiation grade, tumour stage, vessel invasion, neural invasion, and Ki67 expression. Gross tumour classification was divided into infiltration type, mass type, and ulceration type. Histological type of carcinoma was divided into classical adenocarcinoma, mucinous adenocarcinoma, and signet ring cell carcinoma. Tumour differentiation grades were classified as well-differentiated, moderately differentiated, and poorly differentiated. The cut-off value for the number of retrieved lymph nodes (< 12 vs. ≥ 12) was determined as described in the National Comprehensive Cancer Network guidelines [20]. Tumour stage was determined according to the guidelines of the American Joint Committee on Cancer TNM staging system (8th edition) [21]. Ki67 expression was categorized into three groups based on the number of positively-stained tumour cells among all counted tumour cells: low Ki67 (0–10%), moderate Ki67 (10%-25%), and high Ki67 (> 25%) [22]. The expression of MMR proteins, including MLH1, PMS2, MSH2, and MSH6, was analysed using immunohistochemistry testing. Negative expression of one or more MMR proteins was considered a deficient MMR (dMMR) status, whereas positive expression of all four proteins was regarded a proficient MMR (pMMR) status. The primary oncologic outcome was overall survival (OS) rate. OS was defined as the period from the date of diagnosis to the date of death from any cause [23].

### Statistical analysis

Statistical evaluation was performed using the R software, version 4.0.2 (http://www.r-project.org). Normally distributed continuous variables are presented as mean ± standard deviation, whereas non-normal variables are reported as median (interquartile range). The variables were tested for normal distribution using the Shapiro–Wilk normality test. The means of two continuous normally distributed variables were compared using the independent samples student's *t*-test. The Mann–Whitney U test and Kruskal–Wallis test were used to compare the means of two and three or more groups of non-normally distributed variables, respectively. Categorical variables were compared using Pearson’s chi-square test or Fisher’s exact test. Bonferroni correction was applied for post hoc analysis after Chi-squared testing. Time-dependent survival probabilities were calculated using the Kaplan–Meier method, and the log-rank significance test was used to estimate the survival differences among various subgroups. OS was used as the primary outcome measure. The median follow-up duration and its interquartile range were calculated for the entire study cohort using the reverse Kaplan–Meier method. Univariate and multivariate analyses of various clinicopathological variables were performed using Cox's proportional hazards model to identify the independent prognostic factors for OS. Clinical covariates with *P* values < 0.2 in the univariate analysis were used in the multivariate Cox regression [24]. All statistical tests were two-sided, and *P* < 0.05 was considered statistically significant.

## Results

### Clinicopathological characteristics of patients with synchronous colorectal cancer and primary unifocal colorectal cancer

A total of 2341 patients with CRC were included in this study cohort. Of these, 787 patients were excluded because they met the exclusion criteria (Fig. 1). Thus, 1554 patients were included in the analysis. Comparison of the clinicopathologic characteristics of the patients with PCRC with those of patients with SCRC is shown in Table 1. Of these 1554 patients included in this study, 422 had SCRC (27.16%) and 1132 had PCRC (72.84%). The number of elderly patients with SCRC was higher than those with PCRC (*P* < 0.001). Male patients were more common in the SCRC group than in the PCRC group (70.14% vs. 61.31%, *P* = 0.002). More patients with SCRC had a family history of tumours than patients with PCRC (*P* = 0.033). Regarding tumour locations, 110 (26.07%) patients with SCRC had tumours in the rectum, 81 (19.19%) had tumours in the right colon, 93 (22.04%) had tumours in the left colon, and 138 patients (32.70%) had tumours in multiple segments. Patients with SCRC had more advanced disease than those with PCRC (*P* < 0.001). In addition, the SCRC group had a significantly lower proportion of patients with harvested lymph nodes < 12 than the PCRC group (*P* = 0.001). The proportion of patients in the SCRC group with a dMMR status was significantly higher than that in the PCRC group (*P* = 0.001). There were no statistically significant differences in BMI, platelet count, WBC, CA199, CA125, CA153, AFP, and type of ascites between the two groups.

**Table 1:** Clinicopathological characteristics of patients with SCRC and PCRC

	Overall	SCRC	PCRC	*P* value^b^
	N = 1554	N = 422	N = 1132	
Sex (%)				
Female	564 (36.29)	126 (29.86)	438 (38.69)	**0.002**
Male	990 (63.71)	296 (70.14)	694 (61.31)	
EOCRC or LOCRC (%)				
LOCRC	1233 (79.34)	358 (84.83)	875 (77.30)	**0.001**
EOCRC	321 (20.66)	64 (15.17)	257 (22.70)	
Age (yrs) (median [IQR])	61.00 [52.00, 68.00]	63.00 [55.00, 70.75]	60.00 [51.00, 68.00]	**< 0.001**^**a**^
Family history of tumours (%)				
No	1497 (96.33)	399 (94.55)	1098 (97.00)	**0.033**
Yes	57 (3.67)	23 (5.45)	34 (3.00)	
BMI (kg/m^2^) (%)				
18.5 to 24	927 (59.65)	252 (59.72)	675 (59.63)	0.364
< 18.5	152 (9.78)	48 (11.37)	104 (9.19)	
≥ 24	475 (30.57)	122 (28.91)	353 (31.18)	
Hemoglobin (g/L) (%)				
> 110	1063 (68.40)	259 (61.37)	804 (71.02)	**< 0.001**
≤ 100	491 (31.60)	163 (38.63)	328 (28.98)	
Platelet counts (10^9^/L) (%)				
≤ 300	1122 (72.20)	289 (68.48)	833 (73.59)	0.053
> 300	432 (27.80)	133 (31.52)	299 (26.41)	
WBC (10^9^/L) (%)				
≤ 10	1441 (92.73)	388 (91.94)	1053 (93.02)	0.537
> 10	113 (7.27)	34 (8.06)	79 (6.98)	
CEA (ng/mL) (%)				
≤ 10	1157 (74.45)	297 (70.38)	860 (75.97)	**0.029**
> 10	397 (25.55)	125 (29.62)	272 (24.03)	
CA199 (kU/L) (%)				
≤ 27	1225 (78.83)	320 (75.83)	905 (79.95)	0.090
> 27	329 (21.17)	102 (24.17)	227 (20.05)	
CA125 (kU/L) (%)				
≤ 35	1428 (91.89)	383 (90.76)	1045 (92.31)	0.371
> 35	126 (8.11)	39 (9.24)	87 (7.69)	
CA153 (kU/L) (%)				
≤ 25	1526 (98.20)	415 (98.34)	1111 (98.14)	0.965
> 25	28 (1.80)	7 (1.66)	21 (1.86)	
AFP (ng/mL) (%)				
≤ 7	1488 (95.75)	402 (95.26)	1086 (95.94)	0.656
> 7	66 (4.25)	20 (4.74)	46 (4.06)	
Type of ascites (%)				
No	1453 (93.50)	389 (92.18)	1064 (93.99)	0.398
Serous	90 (5.79)	30 (7.11)	60 (5.30)	
Bloody	11 (0.71)	3 (0.71)	8 (0.71)	
Tumor location (%)				
Right colon	321 (20.66)	81 (19.19)	240 (21.20)	**< 0.001**
Left colon	436 (28.06)	93 (22.04)	343 (30.30)	
Rectum	659 (42.41)	110 (26.07)	549 (48.50)	
Multiple segment	138 (8.88)	138 (32.70)	0 (0.00)	
Radical or palliative surgery (%)				
Palliative	185 (11.90)	61 (14.45)	124 (10.95)	0.071
Radical	1369 (88.10)	361 (85.55)	1008 (89.05)	
(y)pTNM staging (%)				
0–I	205 (13.19)	51 (12.09)	154 (13.60)	**0.001**
II	572 (36.81)	130 (30.81)	442 (39.05)	
III	533 (34.30)	152 (36.02)	381 (33.66)	
IV	244 (15.70)	89 (21.09)	155 (13.69)	
Number of retrieved lymph nodes (%)				
< 12	165 (10.62)	26 (6.16)	139 (12.28)	**0.001**
≥ 12	1389 (89.38)	396 (93.84)	993 (87.72)	
Gross classification (%)				
Mass type	583 (37.52)	168 (39.81)	415 (36.66)	0.138
Infiltration type	13 (0.84)	6 (1.42)	7 (0.62)	
Ulceration type	958 (61.65)	248 (58.77)	710 (62.72)	
Histological type (%)				
Classical adenocarcinoma	1452 (93.44)	391 (92.65)	1061 (93.73)	0.663
Mucinous adenocarcinoma	97 (6.24)	29 (6.87)	68 (6.01)	
Signet ring cell carcinoma	5 (0.32)	2 (0.47)	3 (0.27)	
Differentiation grade (%)				
Well differentiated	230 (14.80)	59 (13.98)	171 (15.11)	0.404
Moderately differentiated	1096 (70.53)	293 (69.43)	803 (70.94)	
Poorly differentiated	228 (14.67)	70 (16.59)	158 (13.96)	
Vessel invasion (%)				
Negative	1294 (83.27)	339 (80.33)	955 (84.36)	0.069
Positive	260 (16.73)	83 (19.67)	177 (15.64)	
Neural invasion (%)				
Negative	1243 (79.99)	339 (80.33)	904 (79.86)	0.892
Positive	311 (20.01)	83 (19.67)	228 (20.14)	
Ki67 expression (%)				
Low	95 (6.11)	28 (6.64)	67 (5.92)	0.616
Moderate	204 (13.13)	60 (14.22)	144 (12.72)	
High	1255 (80.76)	334 (79.15)	921 (81.36)	
MMR status (%)				
pMMR	1424 (91.63)	370 (87.68)	1054 (93.11)	**0.001**
dMMR	130 (8.37)	52 (12.32)	78 (6.89)	
Alive status (%)				
Alive	1293 (83.20)	338 (80.09)	955 (84.36)	0.054
Death	261 (16.80)	84 (19.91)	177 (15.64)	

^a^Independent samples Mann–Whitney U test; ^b^The bolded *P* value was statistically significant (*P* < 0.05)

**Fig. 2:**
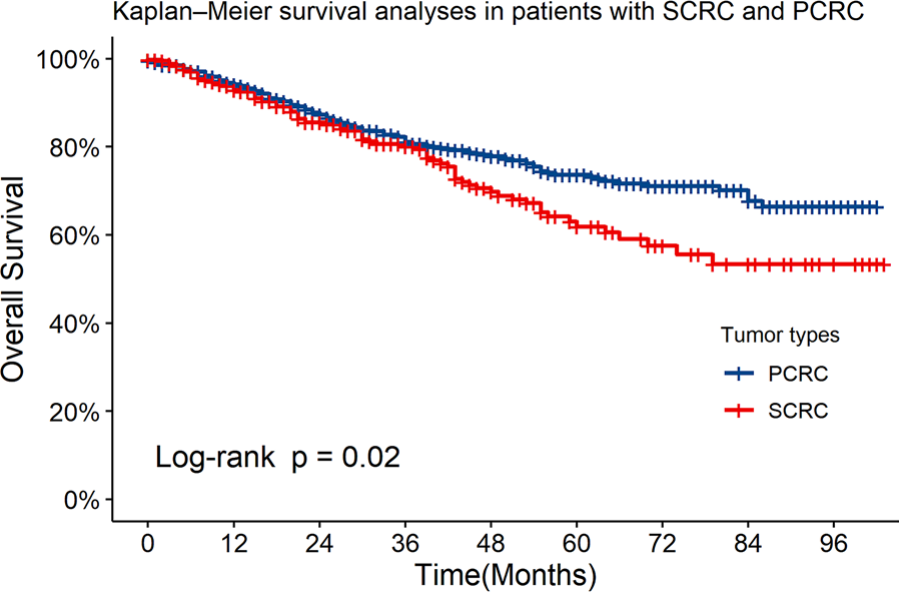
The overall survival (OS) rate in patients with SCRC and those with PCRC. There was a statistically significant difference in 5-year survival between patients with SCRC and those with PCRC

The median follow-up duration was 29 months. The 1-, 3-, and 5-year OS rates in the SCRC group were 93.8%, 80.8%, and 70.5%, respectively. Figure 2 shows that patients with SCRC had a significantly worse OS than those with PCRC. The 5-year OS rates for the PCRC and SCRC groups were 73.7% ± 2.0% and 61.9% ± 3.9% respectively (*P* = 0.02). Median OS was not reached for the whole cohort.

### Clinicopathological characteristics of early-onset colorectal cancer

64 (15.17%) patients in the SCRC group and 257 (22.70%) in the PCRC group had EOCRC (*P* = 0.001). Group difference analysis was performed to evaluate which parameter differed between the patients with EOCRC and LOCRC in the PCRC and SCRC groups. In the PCRC group, there were significant differences in sex, family history of tumour, BMI, platelet count, CEA, histological type, and MMR status between the EOCRC and LOCRC subgroups (Table 2). However, in the SCRC group, there were only significant differences in platelet count, AFP, and MMR status between the two subgroups (Table 2). Furthermore, the differences between the EO-SCRC and EO-PCRC subgroups were analysed and are shown in Table 2. The proportion of patients with increased CA199 and AFP was higher in the EO-SCRC subgroup than in the EO-PCRC subgroup (34.38% vs. 20.62%, *P* = 0.031; 14.06% vs. 5.45%, *P* = 0.034, respectively). Patients in the EO-SCRC subgroup had more TNM stage IV (*P* < 0.001) and fewer opportunities for radical surgery (79.69% vs. 92.22%, *P* = 0.007) than those in the EO-PCRC subgroup.

**Table 2:** Clinicopathological characteristics of patients with EOCRC

	LO-PCRC	EO-PCRC	*P* value^a^ (LO-PCRC vs. EO-PCRC)	LO-SCRC	EO-SCRC	*P* value^a^ (LO-SCRC vs. EO-SCRC)	*P* value^a^ (EO-PCRC vs. EO-SCRC)
	N = 875	N = 257		N = 358	N = 64		
Sex (%)							
Female	322 (36.80)	116 (45.14)	**0.019**	104 (29.05)	22 (34.38)	0.478	0.157
Male	553 (63.20)	141 (54.86)		254 (70.95)	42 (65.62)		
Family history of tumours (%)							
No	854 (97.60)	244 (94.94)	**0.047**	341 (95.25)	58 (90.62)	0.229	0.311
Yes	21 (2.40)	13 (5.06)		17 (4.75)	6 (9.38)		
BMI (kg/m^2^) (%)							
18.5 to 24	539 (61.60)	136 (52.92)	**0.041**	213 (59.50)	39 (60.94)	0.639	0.257
< 18.5	75 (8.57)	29 (11.28)		39 (10.89)	9 (14.06)		
≥ 24	261 (29.83)	92 (35.80)		106 (29.61)	16 (25.00)		
Hemoglobin (g/L) (%)							
> 110	630 (72.00)	174 (67.70)	0.209	223 (62.29)	36 (56.25)	0.439	0.115
≤ 100	245 (28.00)	83 (32.30)		135 (37.71)	28 (43.75)		
Platelet counts (10^9^/L) (%)							
≤ 300	661 (75.54)	172 (66.93)	**0.008**	254 (70.95)	35 (54.69)	**0.015**	0.092
> 300	214 (24.46)	85 (33.07)		104 (29.05)	29 (45.31)		
WBC (10^9^/L) (%)							
≤ 10	817 (93.37)	236 (91.83)	0.475	330 (92.18)	58 (90.62)	0.864	0.953
> 10	58 (6.63)	21 (8.17)		28 (7.82)	6 (9.38)		
CEA (ng/mL) (%)							
≤ 10	645 (73.71)	215 (83.66)	**0.001**	249 (69.55)	48 (75.00)	0.465	0.153
> 10	230 (26.29)	42 (16.34)		109 (30.45)	16 (25.00)		
CA199 (kU/L) (%)							
≤ 27	701 (80.11)	204 (79.38)	0.864	278 (77.65)	42 (65.62)	0.056	**0.031**
> 27	174 (19.89)	53 (20.62)		80 (22.35)	22 (34.38)		
CA125 (kU/L) (%)							
≤ 35	806 (92.11)	239 (93.00)	0.739	329 (91.90)	54 (84.38)	0.093	0.052
> 35	69 (7.89)	18 (7.00)		29 (8.10)	10 (15.62)		
CA153 (kU/L) (%)							
≤ 25	860 (98.29)	251 (97.67)	0.700	354 (98.88)	61 (95.31)	0.126	0.550
> 25	15 (1.71)	6 (2.33)		4 (1.12)	3 (4.69)		
AFP (ng/mL) (%)							
≤ 7	843 (96.34)	243 (94.55)	0.272	347 (96.93)	55 (85.94)	**0.001**	**0.034**
> 7	32 (3.66)	14 (5.45)		11 (3.07)	9 (14.06)		
Type of ascites (%)							
No	816 (93.26)	248 (96.50)	0.157	330 (92.18)	59 (92.19)	0.745	0.208
Serous	52 (5.94)	8 (3.11)		25 (6.98)	5 (7.81)		
Bloody	7 (0.80)	1 (0.39)		3 (0.84)	0 (0.00)		
Tumor location (%)							
Right colon	178 (20.34)	62 (24.12)	0.405	65 (18.16)	16 (25.00)	0.539	**< 0.001**
Left colon	266 (30.40)	77 (29.96)		82 (22.91)	11 (17.19)		
Rectum	431 (49.26)	118 (45.91)		94 (26.26)	16 (25.00)		
Multiple segment	–	–		117 (32.68)	21 (32.81)		
Radical or palliative surgery (%)							
Radical	771 (88.11)	237 (92.22)	0.082	310 (86.59)	51 (79.69)	0.210	**0.007**
Palliative	104 (11.89)	20 (7.78)		48 (13.41)	13 (20.31)		
(y)pTNM staging (%)							
0–I	117 (13.37)	37 (14.40)	0.214	46 (12.85)	5 (7.81)	0.129	**< 0.001**
II	336 (38.40)	106 (41.25)		110 (30.73)	20 (31.25)		
III	292 (33.37)	89 (34.63)		133 (37.15)	19 (29.69)		
IV	130 (14.86)	25 (9.73)		69 (19.27)	20 (31.25)		
Number of retrieved lymph nodes (%)							
< 12	760 (86.86)	233 (90.66)	0.127	334 (93.30)	62 (96.88)	0.415	0.169
≥ 12	115 (13.14)	24 (9.34)		24 (6.70)	2 (3.12)		
Gross classification (%)							
Mass type	324 (37.03)	91 (35.41)	0.763	141 (39.39)	27 (42.19)	0.395	0.064
Infiltration type	6 (0.69)	1 (0.39)		4 (1.12)	2 (3.12)		
Ulceration type	545 (62.29)	165 (64.20)		213 (59.50)	35 (54.69)		
Histological type (%)							
Classical adenocarcinoma	829 (94.74)	232 (90.27)	**0.001**	333 (93.02)	58 (90.62)	0.583	0.675
Mucinous adenocarcinoma	46 (5.26)	22 (8.56)		23 (6.42)	6 (9.38)		
Signet ring cell carcinoma	0 (0.00)	3 (1.17)		2 (0.56)	0 (0.00)		
Differentiation grade (%)							
Well differentiated	129 (14.74)	42 (16.34)	0.163	46 (12.85)	13 (20.31)	0.143	0.557
Moderately differentiated	632 (72.23)	171 (66.54)		255 (71.23)	38 (59.38)		
Poorly differentiated	114 (13.03)	44 (17.12)		57 (15.92)	13 (20.31)		
Vessel invasion (%)							
Negative	735 (84.00)	220 (85.60)	0.600	287 (80.17)	52 (81.25)	0.976	0.502
Positive	140 (16.00)	37 (14.40)		71 (19.83)	12 (18.75)		
Neural invasion (%)							
Negative	695 (79.43)	209 (81.32)	0.564	289 (80.73)	50 (78.12)	0.755	0.687
Positive	180 (20.57)	48 (18.68)		69 (19.27)	14 (21.88)		
Ki67 expression (%)							
Low	48 (5.49)	19 (7.39)	0.469	23 (6.42)	5 (7.81)	0.680	0.982
Moderate	114 (13.03)	30 (11.67)		53 (14.80)	7 (10.94)		
High	713 (81.49)	208 (80.93)		282 (78.77)	52 (81.25)		
MMR status (%)							
pMMR	837 (95.66)	217 (84.44)	**< 0.001**	321 (89.66)	49 (76.56)	**0.006**	0.190
dMMR	38 (4.34)	40 (15.56)		37 (10.34)	15 (23.44)		
Alive status (%)							
Alive	718 (82.06)	237 (92.22)	**< 0.001**	283 (79.05)	55 (85.94)	0.271	0.185
Death	157 (17.94)	20 (7.78)		75 (20.95)	9 (14.06)		

^a^The bolded *P* value was statistically significant (*P* < 0.05)

**Fig. 3:**
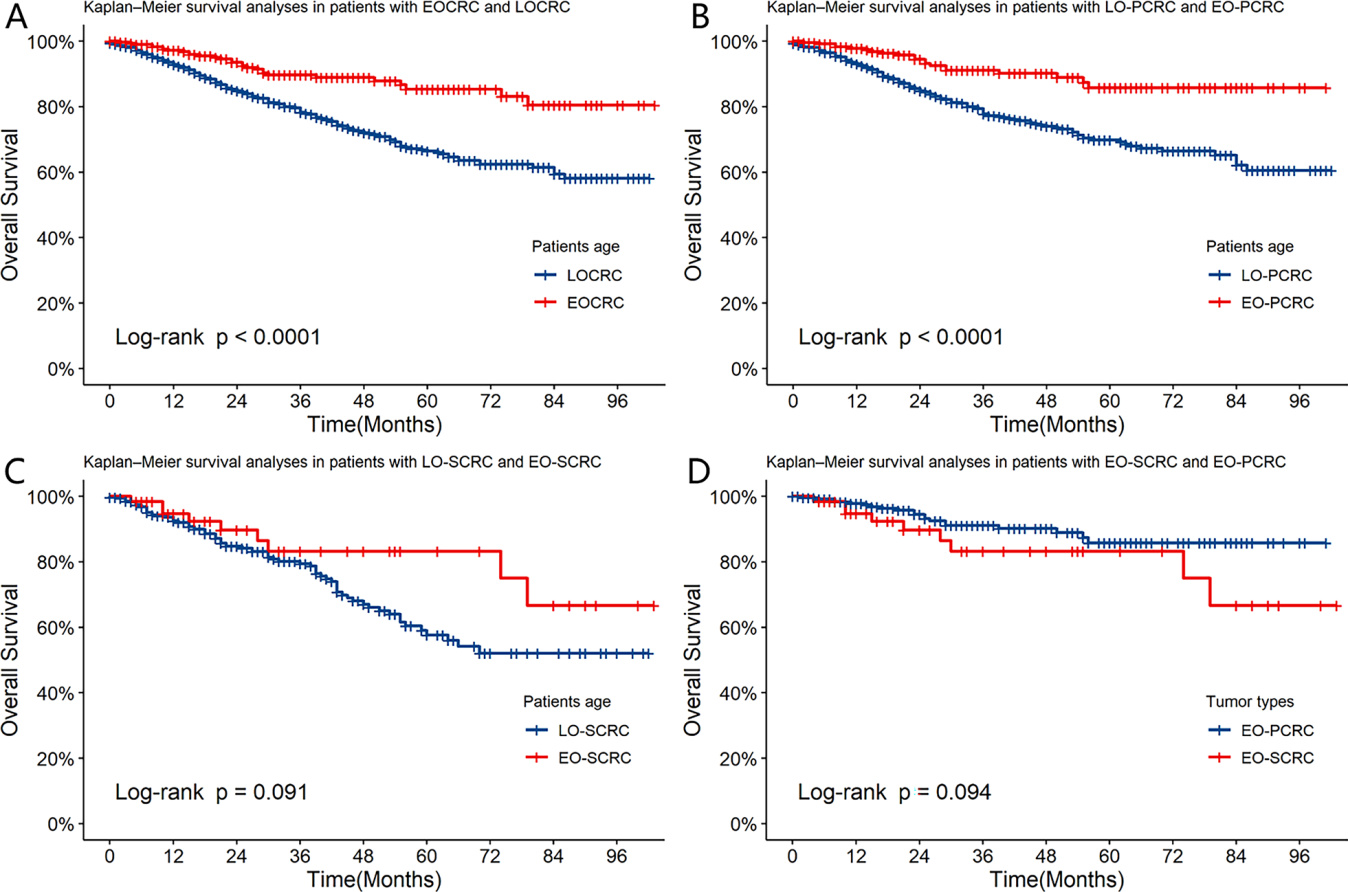
The overall survival (OS) rate in patients with LOCRC and EOCRC in the SCRC and PCRC groups. **A** OS between LOCRC and EOCRC, **B** OS between LO-PCRC and EO-PCRC, **C** OS between LO-SCRC and EO-SCRC, **D** OS between EO-PCRC and EO-SCRC. There was no significant difference in five-year OS between the LO-SCRC and EO-SCRC subgroups and between the EO-PCRC and EO-SCRC subgroups. However, there was a significant difference in five-year OS between the LOCRC and EOCRC subgroups and between the LO-PCRC and EO-PCRC subgroups

During the follow-up period, 20 of 257 patients with EO-PCRC (7.78%) and 9 of 65 patients with EO-SCRC (14.06%) died. The Kaplan–Meier survival analysis showed that there was no significant difference in 5-year OS between the EO-SCRC and LO-SCRC subgroups (83.3% ± 6.03% vs. 57.7% ± 2.74%, *P* = 0.091) (Fig. 3C). In addition, there was no significant difference in 5-year OS between the EO-SCRC and EO-PCRC subgroups (83.3% ± 6.03% vs. 85.7% ± 3.36%, *P* = 0.094) (Fig. 3D). However, there was a significant difference in 5-year OS between the EOCRC and LOCRC subgroups (85.3% ± 2.93% vs. 66.4% ± 2.14%, *P* < 0.001) (Fig. 3A) and between the EO-PCRC and late-onset primary unifocal colorectal cancer (LO-PCRC) subgroups (85.7% ± 3.36% vs. 69.8% ± 2.36%, *P* < 0.001) (Fig. 3B).

### Prognostic factors for synchronous colorectal cancer and primary unifocal colorectal cancer

A univariate analysis was performed to investigate the prognostic factors for CRC. The results showed that EOCRC or LOCRC, family history of tumours, BMI, haemoglobin, CEA, CA199, CA125, and CA153 levels, type of ascites, tumour location, radical or palliative surgery, number of retrieved lymph nodes, gross tumour classification, tumour differentiation grade, vessel invasion; neural invasion, MMR status, and tumour type had a significant effect on 5-year OS (Table 3). Furthermore, ten independent prognostic factors for OS were identified in the multivariate analysis (Table 3). EOCRC had significantly protective effects on the outcome, whereas SCRC did not.

**Table 3:** Prognostic factors for overall survival of patient with CRC

Characteristics	Univariate analysis		Multivariate analysis	
	HR (95%CI)	*P* value^a^	HR (95%CI)	*P* value^a^
Gender				
Female	1	(Reference)		
Male	1.17 (0.90–1.51)	0.235		
EOCRC or LOCRC				
LOCRC	1	(Reference)	1	(Reference)
EOCRC	0.40 (0.27–0.59)	**< 0.001**	0.35 (0.24–0.53)	**< 0.001**
Family history of tumours				
No	1	(Reference)	1	(Reference)
Yes	0.34 (0.12–0.90)	**0.030**	0.56 (0.20–1.53)	0.257
BMI (kg/m^2^)				
18.5 to 24	1	(Reference)	1	(Reference)
< 18.5	1.59 (1.12–2.27)	**0.009**	1.82 (1.26–2.62)	**0.001**
≥ 24	0.86 (0.65–1.15)	0.314	1.05 (0.78–1.41)	0.762
Haemoglobin (g/L)				
> 110	1	(Reference)	1	(Reference)
≤ 100	1.62 (1.26–2.07)	**< 0.001**	1.38 (1.05–1.81)	**0.022**
Platelet counts (10^9^/L)				
≤ 300	1	(Reference)		
> 300	1.12 (0.85–1.47)	0.414		
WBC (10^9^/L)				
≤ 10	1	(Reference)		
> 10	0.94 (0.59–1.49)	0.783		
CEA (ng/mL)				
≤ 10	1	(Reference)	1	(Reference)
> 10	2.84 (2.22–3.64)	**< 0.001**	1.47 (1.11–1.94)	**0.006**
CA199 (kU/L)				
≤ 27	1	(Reference)	1	(Reference)
> 27	2.58 (2.01–3.32)	**< 0.001**	1.22 (0.91–1.62)	0.186
CA125 (kU/L)				
≤ 35	1	(Reference)	1	(Reference)
> 35	3.94 (2.89–5.36)	**< 0.001**	1.95 (1.36–2.79)	**< 0.001**
CA153 (kU/L)				
≤ 25	1	(Reference)	1	(Reference)
> 25	2.94 (1.51–5.73)	**0.002**	2.31 (1.13–4.73)	**0.022**
AFP (ng/mL)				
≤ 7	1	(Reference)		
> 7	0.81 (0.41–1.57)	0.527		
Type of ascites				
No	1	(Reference)	1	(Reference)
Serous	1.53 (1.00–2.35)	0.051	0.72 (0.45–1.16)	0.179
Bloody	5.40 (2.67–10.94)	**< 0.001**	2.86 (1.29–6.34)	**0.010**
Tumor location				
Right colon	1	(Reference)	1	(Reference)
Left colon	0.66 (0.46–0.93)	**0.018**	0.70 (0.48–1.02)	0.067
Rectum	0.74 (0.54–1.01)	0.057	1.02 (0.72–1.44)	0.909
Multiple segment	0.85 (0.54–1.35)	0.496	0.76 (0.44–1.32)	0.326
Radical or palliative surgery				
Radical	1	(Reference)	1	(Reference)
Palliative	9.49 (7.37–12.23)	**< 0.001**	3.99 (2.42–6.56)	**< 0.001**
(y)pTNM staging				
0–I	1	(Reference)	1	(Reference)
II	0.92 (0.63–1.35)	0.672	0.70 (0.48–1.02)	0.067
III	0.82 (0.56–1.21)	0.314	1.02 (0.72–1.44)	0.908
IV	1.02 (0.66–1.56)	0.933	0.76 (0.44–1.32)	0.326
Number of retrieved lymph nodes				
< 12	1	(Reference)	1	(Reference)
≥ 12	1.57 (1.14–2.16)	**0.006**	1.43 (1.02–2.00)	**0.041**
Gross classification				
Mass type	1	(Reference)	1	(Reference)
Infiltration type	3.05 (1.24–7.51)	**0.015**	1.14 (0.40–3.23)	0.802
Ulceration type	1.06 (0.82–1.37)	0.655	0.82 (0.62–1.08)	0.160
Histological type				
Classical adenocarcinoma	1	(Reference)		
Mucinous adenocarcinoma	1.03 (0.65–1.62)	0.915		
Signet ring cell carcinoma	0 (0-Inf)	0.990		
Differentiation grade				
Well differentiated	1	(Reference)	1	(Reference)
Moderately differentiated	1.26 (0.91–1.77)	0.167	1.06 (0.75–1.49)	0.759
Poorly differentiated	1.93 (1.29–2.89)	**0.001**	1.30 (0.83–2.04)	0.252
Vessel invasion				
Negative	1	(Reference)	1	(Reference)
Positive	2.62 (1.98–3.48)	**< 0.001**	1.44 (1.03–2.01)	**0.033**
Neural invasion				
Negative	1	(Reference)	1	(Reference)
Positive	2.07 (1.56–2.73)	**< 0.001**	1.04 (0.75–1.43)	0.824
Ki67 expression				
Low	1	(Reference)		
Moderate	0.81 (0.48–1.37)	0.429		
High	0.84 (0.54–1.32)	0.459		
MMR status				
pMMR	1	(Reference)	1	(Reference)
dMMR	0.45 (0.25–0.80)	**0.007**	0.67 (0.36–1.27)	0.221
Tumor type				
PCRC	1	(Reference)	1	(Reference)
SCRC	1.36 (1.05–1.76)	**0.021**	1.02 (0.75–1.40)	0.889

*HR* hazard ratio, *CI* confidence interval. ^a^The bolded *P* value was statistically significant (*P* < 0.05)

The results of the univariate analysis of the patients with SCRC showed that factors associated with a worse prognosis and OS were decreased haemoglobin level, increased CEA, CA199, CA125, and CA153 levels, bloody ascites, palliative treatment, TNM stages III and IV, positive vessel invasion, and positive neural invasion (Table 4). The multivariable analysis showed that EOCRC, decreased haemoglobin level, increased CA125 level, palliative treatment, and TNM stages III and IV were independent prognostic factors for OS (Table 4).

**Table4:** Prognostic factors for overall survival of patient with SCRC

Characteristics	Univariate analysis		Multivariate analysis	
	HR (95%CI)	*P* value^a^	HR (95%CI)	*P* value^a^
Sex				
Female	1	(Reference)		
Male	1.30 (0.79–2.12)	0.305		
EOCRC or LOCRC				
LOCRC	1	(Reference)	1	(Reference)
EOCRC	0.55 (0.28–1.11)	0.096	0.28 (0.13–0.62)	**0.002**
Family history of tumours				
No	1	(Reference)	1	(Reference)
Yes	0.37 (0.09–1.49)	0.160	0.57 (0.12–2.80)	0.488
BMI (kg/m^2^)				
18.5 to 24	1	(Reference)		
< 18.5	0.97 (0.52–1.83)	0.927		
≥ 24	0.78 (0.47–1.28)	0.320		
Haemoglobin (g/L)				
> 110	1	(Reference)	1	(Reference)
≤ 100	1.80 (1.17–2.77)	**0.007**	1.75 (1.07–2.84)	**0.025**
Platelet counts (10^9^/L)				
≤ 300	1	(Reference)		
> 300	1.20 (0.76–1.89)	0.431		
WBC (10^9^/L)				
≤ 10	1	(Reference)		
> 10	1.19 (0.60–2.38)	0.622		
CEA (ng/mL)				
≤ 10	1	(Reference)	1	(Reference)
> 10	2.65 (1.72–4.09)	**< 0.001**	1.38 (0.82–2.31)	0.220
CA199 (kU/L)				
≤ 27	1	(Reference)	1	(Reference)
> 27	2.24 (1.44–3.47)	**< 0.001**	1.04 (0.61–1.78)	0.884
CA125 (kU/L)				
≤ 35	1	(Reference)	1	(Reference)
> 35	5.88 (3.49–9.91)	**< 0.001**	4.19 (2.23–7.85)	**< 0.001**
CA153 (kU/L)				
≤ 25	1	(Reference)	1	(Reference)
> 25	5.78 (1.79–18.69)	**0.003**	3.65 (0.98–13.57)	0.054
AFP (ng/mL)				
≤ 7	1	(Reference)		
> 7	0.68 (0.22–2.16)	0.516		
Type of ascites				
No	1	(Reference)	1	(Reference)
Serous	1.28 (0.62–2.66)	0.505	0.55 (0.24–1.24)	0.150
Bloody	6.78 (2.12–21.74)	**0.001**	3.53 (0.87–14.27)	0.077
Tumor location				
Right colon	1	(Reference)		
Left colon	0.68 (0.35–1.32)	0.257		
Rectum	0.77 (0.42–1.41)	0.398		
Multiple segment	0.68 (0.38–1.23)	0.200		
Radical or palliative surgery				
Radical	1	(Reference)	1	(Reference)
Palliative	6.45 (4.17–9.96)	**< 0.001**	2.87 (1.17–7.02)	**0.021**
(y)pTNM staging				
0–I	1	(Reference)	1	(Reference)
II	3.13 (0.72–13.6)	0.129	2.77 (0.63–12.22)	0.178
III	5.32 (1.26–22.53)	**0.023**	5.34 (1.24–22.98)	**0.025**
IV	17.27 (4.18–71.47)	**< 0.001**	5.89 (1.16–29.77)	**0.032**
Number of retrieved lymph nodes				
< 12	1	(Reference)	1	(Reference)
≥ 12	1.82 (0.94–3.53)	0.075	1.55 (0.75–3.22)	0.240
Gross classification				
Mass type	1	(Reference)		
Infiltration type	1.75 (0.53–5.71)	0.355		
Ulceration type	0.86 (0.55–1.34)	0.499		
Histological type				
Classical adenocarcinoma	1	(Reference)		
Mucinous adenocarcinoma	0.64 (0.26–1.57)	0.328		
Signet ring cell carcinoma	0 (0-Inf)	0.994		
Differentiation grade				
Well differentiated	1	(Reference)		
Moderately differentiated	1.15 (0.65–2.02)	0.634		
Poorly differentiated	1.09 (0.53–2.23)	0.824		
Vessel invasion				
Negative	1	(Reference)	1	(Reference)
Positive	1.82 (1.11–2.99)	**0.018**	1.09 (0.60–1.98)	0.780
Neural invasion				
Negative	1	(Reference)	1	(Reference)
Positive	2.37 (1.45–3.87)	**0.001**	1.32 (0.71–2.44)	0.378
Ki67 expression				
Low	1	(Reference)	1	(Reference)
Moderate	0.64 (0.28–1.43)	0.275	1.10 (0.45–2.66)	0.833
High	0.58 (0.30–1.13)	0.111	1.00 (0.47–2.13)	0.999
MMR status				
pMMR	1	(Reference)	1	(Reference)
dMMR	0.48 (0.22–1.04)	0.063	0.77 (0.32–1.83)	0.551

*HR* hazard ratio, *CI* confidence interval. ^a^The bolded *P* value was statistically significant (*P* < 0.05)

## Discussion

This study involved a comprehensive analysis of the clinical, histopathological, molecular biological, and follow-up characteristics of a large cohort of patients with PCRC or SCRC. The results of the study showed that there are differences between SCRC and PCRC. In addition, we also noted differences in the clinical features of EOCRC in SCRC compared to LOCRC in SCRC or EOCRC in PCRC. These findings could contribute to better recognition, prevention, and treatment of SCRC and EOCRC.

The characteristics and prognostic factors of PCRC and EOCRC have been widely studied and discussed. However, SCRC, as a special type of CRC with low incidence, is still not completely understood. Thus, its characteristics and prognosis merit further investigation. Furthermore, it is well recognised that certain characteristics of EOCRC differ from those of LOCRC. Consideration of these differences is important for judging prognosis and choosing treatment options. However, EO-SCRC has received little attention in the current research.

The results of this study revealed that the SCRC group had more male and significantly older patients than the PCRC group. Most previous studies have shown that the average age of patients with SCRC is older than that of patients with PCRC. According to a previous review, the mean age of patients with SCRC is 63 years old, which is similar to that of patients with PCRC [2].

Although patients with hereditary colorectal cancer syndromes were excluded from this study, the SCRC group had a higher percentage of patients with a family history of tumours than the PCRC group. Therefore, clinical assessment should focus on the possible existence of SCRC in patients with a family history of tumours. Notably, the SCRC group had a more TNM stage IV than that of the PCRC group. Patients who have tumours with late TNM stages usually have a poor prognosis, which may explain why the survival of patients with SCRC was worse than that of patients with PCRC in the Kaplan–Meier survival analysis. However, in the study by Barz et al., no significant differences in survival were found between patients with SCRC and those with PCRC [25].

The preoperative haematological markers of SCRC, including preoperative tumour markers and routine blood indexes, were analysed in this study. Because of the higher proportion of patients with TNM stage IV in the SCRC group, it was more likely to lost haemoglobin and produce CEA. This can partly explain the differences in CEA and haemoglobin between the SCRC and PCRC groups. Although differences in gross tumour classification, histological type, and tumour differentiation grade between SCRC and PCRC have been reported in previous studies, these differences were not observed in our cohort [25–27]. Consistent with other studies, our results showed that the dMMR status ratio of the SCRC group was higher than that of the PCRC group [28]. MSI is considered to be one of the key causes of CRC [29]. This may account for the relatively high proportion of patients with a dMMR status in the SCRC group.

Although the pathological and epidemiological characteristics of SCRC have been reported in several studies, there are only a few studies on the characteristics of EO-SCRC. In the present study, the proportion of patients in the SCRC group with EOCRC defined using the age-based criteria was lower than that in the PCRC group. Our results showed differences in sex, family history of tumours, BMI, platelet count, CEA level, tumour differentiation grade, and MMR status between the EO-PCRC and LO-PCRC subgroups. These differences have been reported in previous studies as well [12, 30]. However, the EO-SCRC subgroup only differed from the LO-SCRC subgroup in platelet count, AFP, and MMR status. The difference between the EO-SCRC and LO-SCRC subgroups in our study was not as great as that in the PCRC group. Notably, the proportions of patients in the EO-PCRC and EO-SCRC subgroups with a dMMR status were high, a finding that is consistent with the relatively high level of MSI in EOCRC reported in previous studies [12]. Moreover, the proportion of patients with a dMMR status in the EO-SCRC subgroup was higher than that in the EO-PCRC subgroup, which suggested that dMMR status may be more prone to cause multiple primary tumours among younger people [13], although there was not statistically significant difference. We also noted that EO-SCRC subgroup had higher proportion of patients with TNM stage IV, and less chance to undergo radical surgery compared with EO-PCRC subgroup. However, survival analysis did not show differences in survival rates between the two groups.

The Kaplan–Meier survival analysis showed that the survival rate of patients with SCRC was worse than that of patients with PCRC. Nevertheless, multivariate Cox analysis showed that SCRC was not an independent prognostic factor for predicting OS. Nosho et al. demonstrated that differences in survival rates exist between SCRC and PCRC, and that SCRC is an independent risk factor that affects prognosis [5]. However, Barz et al. found no significant difference in cause-specific survival and recurrence-free survival between SCRC and PCRC, and reported that SCRC is not an independent prognostic factor [25]. These conflicting results may be due to differences in study designs or limited sample size/power. Another reason may be that SCRC triggers other factors that could affect prognosis. More evidence is needed to clarify the association between SCRC and prognosis.

The correlation between EOCRC and prognosis remains controversial [31]. Some studies have shown that EOCRC has a poor prognosis [32], whereas others showed similar or better prognoses than that reported for LOCRC [33, 34]. In the present study, patients with EOCRC, both in the entire CRC cohort and in the SCRC group, had better prognoses than those with LOCRC. This could possibly be due to the fact that some favourable characteristics of EOCRC outweigh its unfavourable characteristics. For example, the EO-SCRC subgroup in the present study had a high proportion of patients with a dMMR status. Patients with a dMMR status always have better prognoses. This may explain why patients with EO-SCRC have better prognoses. Similar results were observed in the EO-PCRC cohort as well.

The main limitation of this study is its design. This was a single-centre, retrospective, observational study, which runs the risk for patient selection bias and group selection bias. The study cohort was collected from one of the main clinical centers for CRC in China, which many patients, especially the complicated cases, were referred to this hospital. And the inclusion criteria are different to other literature, which only the operated CRC cases were included and analyzed. It could be the reason for the higher proportion of SCRC in the study. In addition, the cases with incomplete data were excluded for exclusion criteria, which may introduce an element of selection bias. Despite these limitation, the results obtained from this study are reliable because strict and clear inclusion and exclusion criteria were used in this study and a large number of cases were included. Furthermore, we focused on sporadic SCRC; therefore, hereditary colorectal cancer syndromes were excluded as much as possible using clinical data.

## Conclusion

This study showed that there are several differences between PCRC and SCRC among the patients with operative treatment. In addition, it showed that EO-SCRC is a subgroup different from LO-SCRC and EO-PCRC. Furthermore, the clinical features and prognostic factors of CRC and SCRC were identified in this study. Patients with SCRC showed a poorer survival rate than those with PCRC. However, we found that SCRC is not an independent prognostic factor for CRC, whereas EOCRC is a protective factor for the prognosis of CRC and SCRC. Future large multicentre studies are required to corroborate the findings of this study.

## Data Availability

All analyzed data are included in this published article. The original data are available upon reasonable request to the corresponding author.

## Abbreviations

CRC: Colorectal cancer
SCRC: Synchronous colorectal cancer
PCRC: Primary unifocal colorectal cancer
LO-SCRC: Late-onset synchronous colorectal cancer
EO-SCRC: Early-onset synchronous colorectal cancer
EOCRC: Early-onset colorectal cancer
LOCRC: Late-onset colorectal cancer
LO-PCRC: Late-onset primary unifocal colorectal cancer
EO-PCRC: Early-onset primary unifocal colorectal cancer
BMI: Body mass index
MSI: Microsatellite instability
dMMR: Deficient mismatch repair proteins
OS: Overall survival
CEA: Carcinogenic antigen
AFP: Alpha-fetoprotein
CA: Cancer antigen
